# Nationwide differences in cytology fixation and processing methods and their impact on interlaboratory variation in PD-L1 positivity

**DOI:** 10.1007/s00428-022-03446-w

**Published:** 2022-11-12

**Authors:** Bregje M. Koomen, Mirthe de Boer, Carmen van Dooijeweert, Anne S. R. van Lindert, Ivette A. G. Deckers, Quirinus J. M. Voorham, Stefan M. Willems

**Affiliations:** 1 Department of Pathology, University Medical Center Utrecht, Utrecht University, Heidelberglaan 100, Utrecht, 3584 CX The Netherlands; 2 Department of Pulmonology, University Medical Center Utrecht, Utrecht University, Heidelberglaan 100, Utrecht, 3584 CX The Netherlands; 3 PALGA Foundation, De Bouw 123, 3991 SZ Houten, The Netherlands; 4 Department of Pathology and Medical Biology, University of Groningen, University Medical Center Groningen, Hanzeplein 1, Groningen, 9713 GZ The Netherlands

**Keywords:** Non-small cell lung cancer, Programmed death ligand-1, Immunocytochemistry, Fixatives, Cytological techniques, Interlaboratory variation

## Abstract

Programmed death ligand-1 (PD-L1) immunostaining, which aids clinicians in decision-making on immunotherapy for non-small cell lung cancer (NSCLC) patients, is sometimes performed on cytological specimens. In this study, differences in cytology fixation and cell block (CB) processing between pathology laboratories were assessed, and the influence of these differences on interlaboratory variation in PD-L1 positivity was investigated. Questionnaires on cytology processing were sent to all Dutch laboratories. Information gathered from the responses was added to data on all Dutch NSCLC patients with a mention of PD-L1 testing in their cytopathology report from July 2017 to December 2018, retrieved from PALGA (the nationwide network and registry of histo- and cytopathology in the Netherlands). Case mix-adjusted PD-L1 positivity rates were determined for laboratories with known fixation and CB method. The influence of differences in cytology processing on interlaboratory variation in PD-L1 positivity was assessed by comparing positivity rates adjusted for differences in the variables fixative and CB method with positivity rates not adjusted for differences in these variables. Twenty-eight laboratories responded to the survey and reported 19 different combinations of fixation and CB method. Interlaboratory variation in PD-L1 positivity was assessed in 19 laboratories. Correcting for differences in the fixative and CB method resulted in a reduction (from eight (42.1%) to five (26.3%)) in the number of laboratories that differed significantly from the mean in PD-L1 positivity. Substantial variation in cytology fixation and CB processing methods was observed between Dutch pathology laboratories, which partially explains the existing considerable interlaboratory variation in PD-L1 positivity.

**Supplementary Information:**

The online version contains supplementary material available at 10.1007/s00428-022-03446-w.

## Introduction

Globally, lung cancer is one of the most frequent forms of cancer, with more than 2.2 million new cases in 2020, accounting for 11.4% of all cancer cases worldwide [1]. Lung cancer also accounts for the most cancer-related deaths worldwide [1], with a 5-year survival rate of only 10–20% for patients diagnosed between 2010 and 2014 in most countries [2]. Non-small cell lung cancer (NSCLC), one of the two main histopathological types (the other being small cell lung cancer), accounts for 85% of lung cancer patients [3, 4]. For patients with advanced NSCLC without actionable mutations in driver genes such as *EGFR* or *ALK*, immunotherapy may prove beneficial and has become part of standard treatment. Patients with stage IV NSCLC may qualify for first-line monotherapy with pembrolizumab, a programmed death receptor-1 (PD-1) inhibitor, when at least 50% of their tumor cells show expression of programmed death ligand-1 (PD-L1) [5, 6]. Similarly, patients with unresectable stage III NSCLC may receive consolidation treatment with durvalumab, a PD-L1 inhibitor, after chemoradiotherapy, for which the European Society for Medical Oncology (ESMO) recommended that patients should have a PD-L1 expression of at least 1% on tumor cells [7].

PD-L1 expression is determined by pathologists through immunohistochemistry, which has been validated on histological specimens. In clinical practice, however, quite often, minimally invasive techniques such as fine needle aspirations (FNA) are used to collect diagnostic material [8]. In those instances, pathologists may be asked to perform PD-L1 immunostaining on cytology samples, for which usually cell blocks (CBs) are prepared. While histological specimens are generally processed into formalin-fixed paraffin-embedded (FFPE) tissue blocks, numerous ways of processing cytology specimens into cell blocks exist [9]. What is more, various non-formaldehyde-based fixatives are used for the fixation of cytological specimens [9, 10]. We and others have shown that the use of alcohol-based fixatives may negatively influence the immunoreactivity of various antibodies [11–14], including PD-L1 [15–17]. We have also demonstrated that a considerable amount of variation in PD-L1 positivity rates exists between pathology laboratories in a real-world setting, both in PD-L1 positivity rates based on histological material and in PD-L1 positivity rates based on cytological material [18]. Besides inter-observer variability between pathologists, pre-analytical factors such as use of different fixatives and CB methods might play a role in causing this variation, too. In this study, the variation in fixation and CB processing of cytology samples between pathology laboratories in the Netherlands was assessed. Subsequently, we investigated whether variation in fixation and processing methods influences interlaboratory variation in PD-L1 positivity, using real-world clinical pathology data of a large cohort of NSCLC patients.

## Materials and methods

### Survey on fixation and CB methods

In order to gather information on methods used for fixation and CB processing of cytology samples in each individual laboratory, a questionnaire was sent to all pathology laboratories in the Netherlands. The questionnaire contained questions on how many different processing methods were used within the individual laboratory, which collection media and fixatives were used for various cytology samples, if post-fixation in a different fixative was part of the process, which fixation times were used, and which method was used for processing the cytology sample into a CB (Supplementary information 1). The respondents were asked to specifically answer these questions for cytological samples from NSCLC patients that may had been tested for PD-L1 between 1 July 2017 and 31 December 2018, corresponding with the study period used for extraction of data from the PALGA data set (see “Data source and data extraction”).

### Data source and data extraction

In order to analyze the impact of fixation and CB methods on interlaboratory variation in PD-L1 positivity, data were extracted from PALGA, the nationwide network and registry of histo- and cytopathology in the Netherlands. The PALGA registry contains all pathology records from all Dutch pathology laboratories since 1991 [19]. All pathology laboratories have given consent for the storage of their data by PALGA, and for the scientific use of these data. Patients can opt out of consenting to the use of their data for research purposes. Since this specific study had a national, non-interventional retrospective design and all data were analyzed anonymously, patient consent was waived. This study was approved by PALGA’s Scientific Council and Privacy Committee, and all data were handled according to the General Data Protection Regulation (GDPR).

Data on all NSCLC patients in the Netherlands with a mention of PD-L1 testing in their pathology report between 1 July 2017 and 31 December 2018 were retrieved. We have reported on this previously in another manuscript [18], in which we assessed interlaboratory variation in PD-L1 positivity in both histological and cytological material on a nationwide level. Patients with multiple primary lung tumors were excluded from the data set, because treatment of one of these tumors could potentially have influenced PD-L1 expression in the other tumor [20, 21]. For the current study, only the data on cytological specimens were used. Since information on fixation and CB method is not part of standard pathology reporting, the information from the survey was used to enrich the data from PALGA. In order to do so, the various methods described by the respondents were divided into categories within two variables, i.e. fixative and CB method. These variables were then added to the PALGA data set. In order to enable the linking of the information gathered from the survey to the data retrieved from PALGA, the survey was sent to laboratories through PALGA, ensuring that the laboratories taking part remained anonymous to the researchers.

From all pathology reports concerning cytological specimens with known fixation and CB method, the following data were extracted: age and sex of the patient, histologic subtype of the tumor, amount of PD-L1 tests performed, source of material for PD-L1 test(s), reported tumor proportion score (TPS), and number of tumor cells present (< 100 or ≥ 100), if reported.

### Analysis of interlaboratory variation in PD-L1 positivity in relation to fixation and CB methods

Equal to what we described in our previous paper [18], variation in PD-L1 positivity was studied by comparing the proportions of reported PD-L1 positive patients between the laboratories that performed PD-L1 testing. PD-L1 positivity was determined according to two clinically relevant cutoffs, i.e., ≥ 1% and ≥ 50%. Analysis of interlaboratory variation was performed for each of these cutoffs separately. Only cytology samples with known fixation and CB method were included. Furthermore, only patients from laboratories that had performed PD-L1 tests in ≥ 30 patients during the study period were included, and for each patient, only one PD-L1 test performed on a cytological sample was included. Patients with discordant results of multiple PD-L1 tests performed on cytological material were excluded, since it was impossible to determine which of the test results could be considered as the “true” result. This concerned results from multiple tests performed on the same tumor focus as well as results from tests on different foci of the same tumor process (e.g., primary tumor and metastasis). Patients with inconclusive test results only and patients with tests without a reported TPS were excluded as well.

Plots displaying interlaboratory variation in PD-L1 positivity in cytological material were created. Information on fixative was incorporated in these plots by using colors to display the fixative that was used most in each laboratory. Some laboratories performed PD-L1 testing for both their own and external laboratories, and fixation and CB methods could also differ within one laboratory, which is why some laboratories performed PD-L1 testing on cytological material fixed in various fixatives. Whenever laboratories used two fixatives in a fairly even distribution (up to 65–35%), two colors were used.

### Statistical analysis

Patient and sample characteristics were summarized using counts and proportions. Differences between PD-L1 positive and negative subgroups were tested by using a Pearson’s *χ*^2^ test for categorical variables and a *t*-test for continuous variables. Potential associations between PD-L1 positivity and fixative or CB method were assessed using univariable logistic regression analysis. PD-L1 positivity was determined using the 1% cutoff and the 50% cutoff, separately.

The mean PD-L1 positivity rate of all patients included was determined for both the 1% cutoff and the 50% cutoff. Differences in PD-L1 positivity rates between laboratories were displayed in funnel plots, which showed the mean PD-L1 positivity rate with its 95% confidence limits and the percentage of PD-L1 positive patients plotted against the total number of patients tested for each laboratory. All laboratories falling outside the 95% confidence limits were considered to differ significantly from the mean.

The PD-L1 positivity rates used in the funnel plots were adjusted for differences in patient and sample characteristics (i.e., case mix) by performing multivariable logistic regression analysis. Based on the multivariable regression model, case mix-adjusted positivity rates were determined by dividing the observed percentage of PD-L1 positive patients per laboratory by the expected percentage, followed by multiplying with the mean percentage of PD-L1 positivity. The predetermined variables that were included in the adjustment analyses were age, sex, histologic subtype, and source of material used for PD-L1 testing. Additional case mix-adjusted PD-L1 positivity rates were calculated using multivariable logistic regression analysis that also included the variables fixative and CB method. These positivity rates were then compared with the case mix-adjusted positivity rates of the same laboratories without the variables fixative and CB method. Furthermore, the likelihood ratio test (LRT) was used to compare the goodness of fit of both multivariable logistic regression models (with and without the variables fixative and CB method).

All statistical analyses were performed using IBM SPSS Statistics version 26.

## Results

### Nationwide variation in fixation and CB methods of cytology samples

**Fig. 1:**
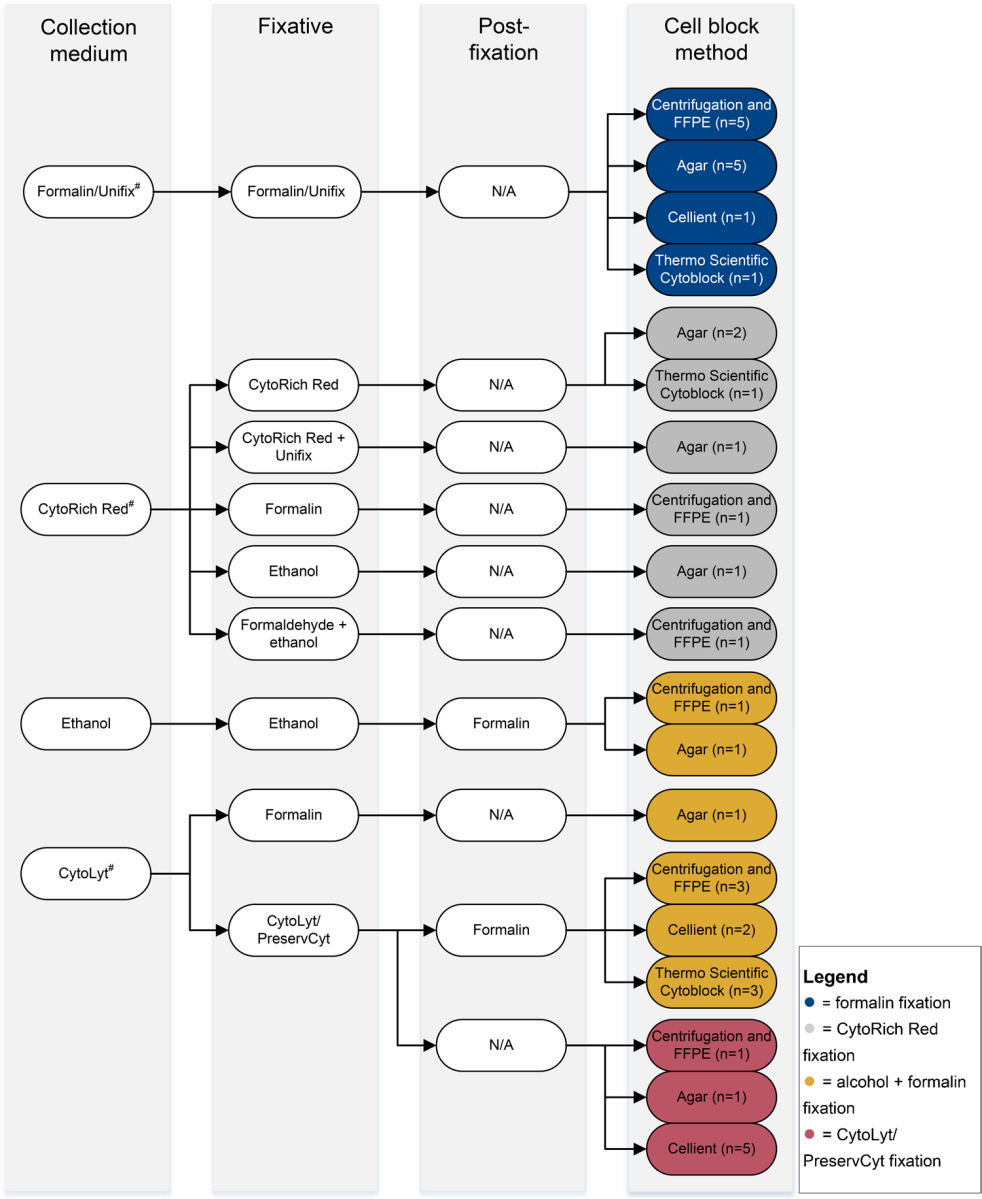
Overview of the various combinations of fixation and cell block methods for cytology samples as described by the survey respondents. The final column displays the number of times each combination is used. Colors depict the overall fixation method for each combination (see legend). ^#^Fluids were not always received in a collection medium, but rather as fresh fluids. Abbreviations: FFPE, formalin-fixed paraffin-embedded; N/A. not applicable

We received responses from 28 (66.7%) of the 42 laboratories to that the questionnaire was sent to. From the responses, 19 different ways of processing cytology samples could be discerned. Figure 1 provides an overview of the various combinations of collection medium, fixative, post-fixation, and CB method, with the number of times each combination was used by a laboratory in the final column. Sometimes different processing methods were used within one laboratory, depending on the type of cytological material. This explains why the numbers in the final column add up to 37 instead of 28. Variation in mean fixation time ranged from 20 min to 36 h, with a mean of 12 h.

Formalin or Unifix, a substance also containing formaldehyde, was used most often in the various combinations of fixatives and CB methods employed by the different laboratories (12 out of 37 times, 32.4%). Alcohol fixation (methanol- or ethanol-based) followed by formalin fixation was used 11 of 37 times (29.7%). Seven (18.9%) of all 37 processing methods contained a step involving CytoRich Red fixation, a solution that contains both alcohols and formaldehyde. Finally, in 7 out of 37 cases (18.9%), the cytological material was fixed using CytoLyt and PreservCyt (Hologic, Marlborough, Massachusetts, USA), both methanol-based, without formalin post-fixation.

Four different CB methods were used, i.e., centrifugation of the cytology sample and processing the cell pellet into an FFPE CB, agar embedding, the Thermo Scientific or Shandon CB method (Thermo Fischer Scientific, Waltham, Massachusetts, USA), and the Cellient-automated CB system (Hologic, Marlborough, Massachusetts, USA). Centrifugation and processing into an FFPE CB and the agar-based method were used most often (12 out of 37 times, 32.4%). The Cellient CB system and Thermo Scientific CB method were used in 8 (21.6%) and 5 (13.5%) out of 37 cases, respectively.

### Patient selection process from the PALGA data set

Information on fixation methods used by the various laboratories was divided into four categories (formalin fixation, CytoRich Red fixation, alcohol (methanol or ethanol) fixation with formalin post-fixation, and CytoLyt/PreservCyt fixation (without formalin post-fixation)) and added to the PALGA data set. Similarly, information on CB methods used by the various laboratories was also divided into four categories (centrifugation and FFPE CB, agar-based CB, Thermo Scientific CB, and Cellient CB) and added to the PALGA data set.

**Fig. 2:**
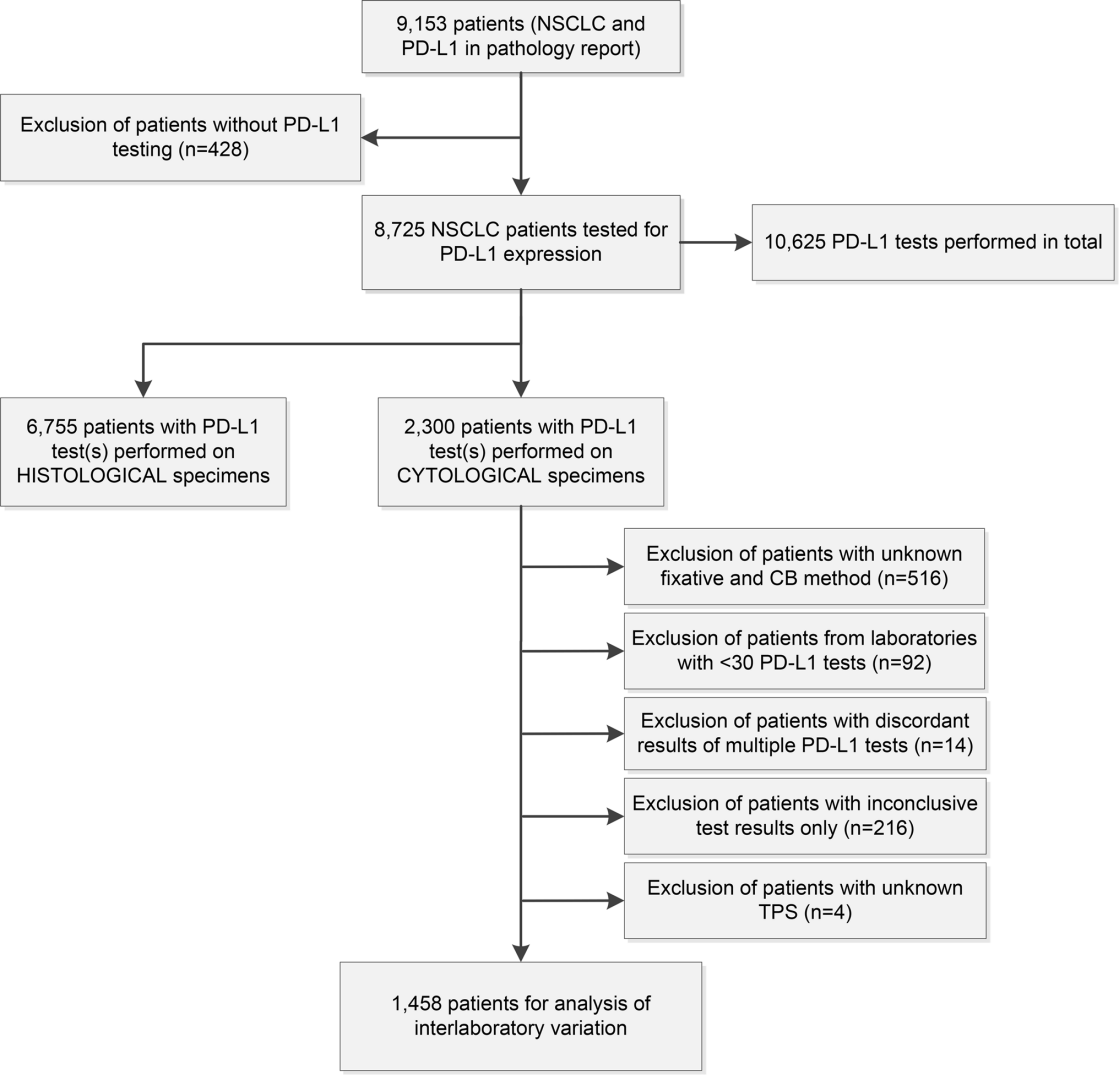
Flowchart of the patient selection process from the PALGA data set. CB, cell block; NSCLC, non-small cell lung cancer; PD-L1, programmed death ligand-1; TPS, tumor proportion score

The PALGA data set showed that during the study period, 10,625 PD-L1 tests were performed in 8725 patients with NSCLC in the Netherlands. Data from 42 laboratories were included, of which 32 performed PD-L1 testing on cytology samples. Of all tests, 2665 (25.1%) were performed on cytological material of 2300 patients. Based on the results from our survey, information on fixation and CB method could be added to the samples of 1784 patients, resulting in the exclusion of 516 patients. After this, 92 patients from laboratories that performed PD-L1 testing in < 30 patients were excluded. Finally, after the exclusion of patients with discordant results from multiple PD-L1 tests (*n* = 14), patients with inconclusive test results only (*n* = 216), and patients with tests with unknown TPS (*n* = 4), 1458 patients from 19 laboratories remained for analysis of interlaboratory variation in PD-L1 positivity (Fig. 2). The patients with inconclusive test results only had a total of 259 tests performed, of which 195 (75.3%) were reported to have an insufficient amount of viable tumor cells (< 100). Characteristics of all included patients and their samples tested for PD-L1 are displayed in Table 1. Proportions significantly differed between PD-L1 positivity and negativity across sex, histologic subtype, fixative, and CB method for both cutoffs.

**Table 1:** Differences in patient and sample characteristics between PD-L1 negativity and PD-L1 positivity (PD-L1 < 1% vs. PD-L1 ≥ 1% and PD-L1 < 50% vs. PD-L1 ≥ 50%)

	Total (*n* = 1458)	PD-L1 < 1% (*n* = 705)	PD-L1 ≥ 1% (*n* = 753)	*p*-value	PD-L1 < 50% (*n* = 1042)	PD-L1 ≥ 50% (*n* = 416)	*p*-value
Age in years (mean (SD))	66.5 (10.1)	66.6 (10.3)	66.5 (9.8)	0.82	66.6 (10.2)	66.3 (9.7)	0.66
Sex							
Male	777 (53.3%)	408 (57.9%)	369 (49.0%)	< 0.01	583 (56.0%)	194 (46.6%)	< 0.01
Female	681 (46.7%)	297 (42.1%)	384 (51.0%)		459 (44.0%)	222 (53.4%)	
Histologic subtype							
AC	1105 (75.8%)	500 (70.9%)	605 (80.3%)	< 0.01	758 (72.7%)	347 (83.4%)	< 0.01
SCC	206 (14.1%)	122 (17.3%)	84 (11.2%)		167 (16.0%)	39 (9.4%)	
NSCLC NOS	132 (9.1%)	73 (10.4%)	59 (7.8%)		105 (10.1%)	27 (6.5%)	
Other	15 (1.0%)	10 (1.4%)	5 (0.7%)		12 (1.2%)	3 (0.7%)	
Source of material							
Primary tumor	81 (5.6%)	41 (5.8%)	40 (5.3%)	0.54	60 (5.8%)	21 (5.0%)	0.64
Metastasis	103 (7.1%)	54 (7.7%)	49 (6.5%)		72 (6.9%)	31 (7.5%)	
Lymph node metastasis	873 (59.9%)	430 (61.0%)	443 (58.8%)		618 (59.3%)	255 (61.3%)	
Pleural effusion	342 (23.5%)	154 (21.8%)	188 (25.0%)		245 (23.5%)	97 (23.3%)	
Bronchial brush/fluid	59 (4.0%)	26 (3.7%)	33 (4.4%)		47 (4.5%)	12 (2.9%)	
Fixative				< 0.01			< 0.05
Formalin	459 (31.5%)	185 (26.2%)	274 (36.4%)		306 (29.4%)	153 (36.8%)	
CytoRich Red	301 (20.6%)	140 (19.9%)	161 (21.4%)		213 (20.4%)	88 (21.2%)	
Alcohol + formalin	346 (23.7%)	175 (24.8%)	171 (22.7%)		255 (24.5%)	91 (21.9%)	
CytoLyt/PreservCyt	352 (24.1%)	205 (29.1%)	147 (19.5%)		268 (25.7%)	84 (20.2%)	
Cell block method				< 0.01			< 0.01
Centrifugation and FFPE	301 (20.6%)	143 (20.3%)	158 (21.0%)		213 (20.4%)	88 (21.2%)	
Agar	627 (43.0%)	255 (36.2%)	372 (49.4%)		417 (40.0%)	210 (50.5%)	
Thermo Scientific Cytoblock	191 (13.1%)	91 (12.9%)	100 (13.3%)		138 (13.2%)	53 (12.7%)	
Cellient	339 (23.3%)	216 (30.6%)	123 (16.3%)		274 (26.3%)	65 (15.6%)	

*AC*, adenocarcinoma; *FFPE*, formalin-fixed paraffin-embedded; *NOS*, not otherwise specified; *NSCLC*, non-small cell lung cancer; *PD-L1*, programmed death ligand-1; *SD*, standard deviation; *SCC*, squamous cell carcinoma

### Analysis of the association between fixative and PD-L1 positivity

Within the different categories of the variable fixative, the mean PD-L1 positivity rate based on the 1% cutoff was highest in the samples fixed in formalin (59.7%). After this came CytoRich Red (53.5%) and alcohol fixation with formalin post-fixation (49.4%), followed by CytoLyt/PreservCyt fixation without formalin post-fixation (41.8%). At the 50% cutoff, the mean percentage of PD-L1 positive cases was 33.3% for all samples fixed in formalin. Mean PD-L1 positivity was 29.2% for samples fixed in CytoRich Red, 26.3% for samples fixed in alcohol with formalin post-fixation, and 23.9% for samples fixed in CytoLyt/PreservCyt (Supplementary Fig. 1a).

Univariable logistic regression analysis revealed a statistically significant association between fixative and PD-L1 positivity for both the 1% cutoff and the 50% cutoff (Table 2), with the odds of scoring PD-L1 as positive being significantly lower in samples fixed in CytoLyt/PreservCyt (without formalin post-fixation) or in alcohol with formalin post-fixation compared with samples fixed in formalin only. There was no significant difference in the odds of scoring PD-L1 as positive between samples fixed in CytoRich Red and those fixed in formalin.

**Table 2:** Univariable logistic regression analysis for assessment of the association between the variables fixative and cell block method and PD-L1 positivity (defined as ≥ 1% and ≥ 50% positive tumor cells)

	PD-L1 ≥ 1% vs. < 1%		PD-L1 ≥ 50% vs. < 50%	
	OR (95% CI)	Overall *p*-value	OR (95% CI)	Overall *p*-value
Fixative		< 0.01		< 0.05
Formalin	1.00 reference		1.00 reference	
CytoRich Red	0.78 (0.58–1.04)		0.83 (0.60–1.13)	
Alcohol + formalin	**0.66 (0.50–0.87)**		**0.71 (0.52–0.97)**	
CytoLyt/PreservCyt	**0.48 (0.37–0.64)**		**0.63 (0.46–0.86)**	
Cell block method		< 0.01		< 0.01
Centrifugation and FFPE	1.00 reference		1.00 reference	
Agar	**1.32 (1.00–1.74)**		1.22 (0.90–1.64)	
Thermo Scientific Cytoblock	1.00 (0.69–1.43)		0.93 (0.62–1.39)	
Cellient	**0.52 (0.38–0.71)**		**0.57 (0.40–0.83)**	

Data in bold indicate a statistically significant difference in the odds of scoring PD-L1 as positive compared to the reference category

*CI*, confidence interval; *FFPE*, formalin-fixed paraffin-embedded; *OR*, odds ratio; *PD-L1*, programmed death ligand-1

### Analysis of the association between CB method and PD-L1 positivity

The mean PD-L1 positivity rate at the 1% cutoff for samples that were centrifuged and processed into FFPE CBs was 52.5%. For the other categories, mean PD-L1 positivity rates were 59.3% (agar CBs), 52.4% (Thermo Scientific CBs), and 36.3% (Cellient CBs). At the 50% cutoff, the mean PD-L1 positivity was 29.2% for samples centrifuged and processed into FFPE CBs. Mean PD-L1 positivity was 33.5% for samples processed into agar CBs, 27.7% for samples processed into Thermo Scientific CBs, and 19.2% for samples processed into Cellient CBs (Supplementary Fig. 1b).

A statistically significant association was found between CB method and PD-L1 positivity for both cutoffs (Table 2). The odds of scoring PD-L1 as positive were significantly lower in samples processed into Cellient CBs in comparison to samples that were centrifuged and processed into FFPE CBs. At the 1% cutoff, the odds of PD-L1 positivity were significantly higher for samples processed into agar-based CBs than for those centrifuged and processed into FFPE CBs. No statistically significant differences in the odds of PD-L1 positivity were found between the Thermo Scientific Cytoblock method and centrifugation and processing into an FFPE CB.

### Fixation and CB methods in relation to interlaboratory variation of PD-L1 positivity

**Fig. 3:**
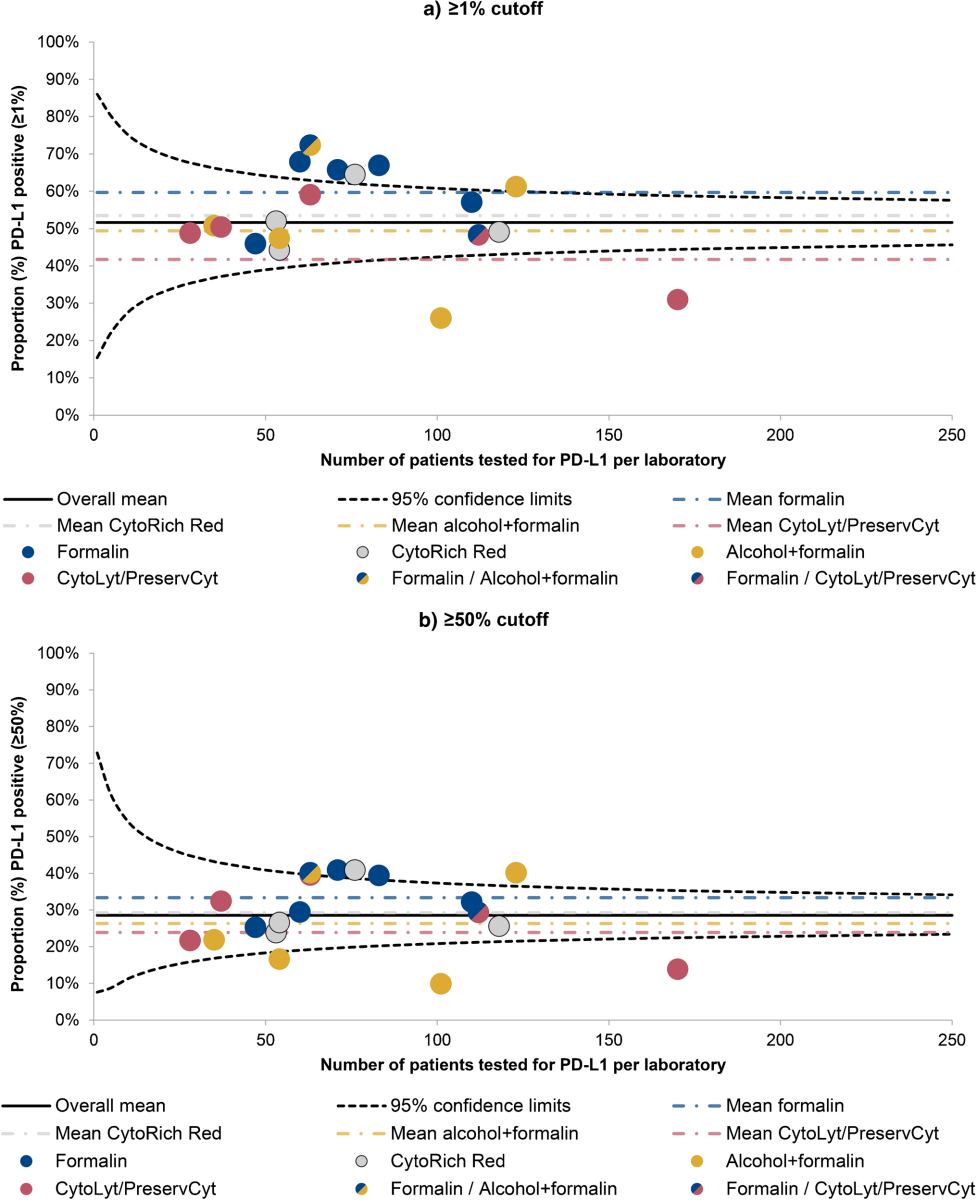
Funnel plots showing interlaboratory variation in programmed death ligand-1 (PD-L1) positivity. PD-L1 positivity was determined using either a 1% cutoff (**a**) or a 50% cutoff (**b**). For each laboratory, case mix-adjusted positivity rates are displayed against the total number of patients tested for PD-L1 (dots). The variables age, sex, histological subtype, and source of material used for PD-L1 testing were included in the case mix adjustment analysis. Colors are used to indicate the fixative that was used the most in each laboratory (see legend). The black line shows the overall mean proportion of PD-L1 positive patients, surrounded by its 95% confidence limits (black dotted lines). The colored dotted lines display the mean PD-L1 positivity rate for each fixative category

The mean PD-L1 positivity rate of all included patients was 51.6% at the 1% cutoff and 28.5% at the 50% cutoff. When positivity rates without any case mix adjustment were plotted against the total number of PD-L1 tests for each laboratory and compared to the overall mean, eight (42.1%) laboratories differed significantly from the mean at the 1% cutoff and nine (47.4%) laboratories differed significantly from the mean at the 50% cutoff (data not shown). After case mix adjustment for sex, age, histologic subtype, and source of material, funnel plots showed eight (42.1%) laboratories differing significantly from the overall mean at both cutoffs (Fig. 3). Case mix adjustment with these variables thus resulted in a reduction of the number of laboratories differing significantly from the mean from nine to eight at the 50% cutoff. No reduction in the number of laboratories differing significantly from the mean was seen at the 1% cutoff. The case mix-adjusted positivity rates from the individual laboratories ranged from 26.0 to 72.4% at the 1% cutoff, and from 9.9 to 40.9% at the 50% cutoff.

When looking at Fig. 3a, attention is drawn to the seven laboratories that used formalin fixation for at least part of their samples (in blue). Five of these laboratories lie above the overall mean, with four laboratories falling outside the upper 95% confidence limit. In contrast, most of the laboratories that primarily used CytoLyt and PreservCyt fixation without formalin post-fixation (in red) lie below the overall mean, with one of the laboratories that fall far below the lower 95% confidence limit also using CytoLyt/PreservCyt fixation. The other laboratory falling below the lower 95% confidence limit mainly used combined alcohol and formalin fixation, but this fixation method was also used by two of the laboratories that lie above the upper 95% confidence limit. Hence, the mean PD-L1 positivity rate of all samples fixed in alcohol followed by formalin post-fixation lies much closer to the overall mean, while the mean of all samples fixed in CytoLyt/PreservCyt without formalin post-fixation lies further below the overall mean. Conversely, the mean of all samples fixed in formalin lies quite far above the overall mean. The differences between the laboratories that used different fixation methods are less apparent at the 50% cutoff (Fig. 3b), although a similar pattern can still be discerned.

**Fig. 4:**
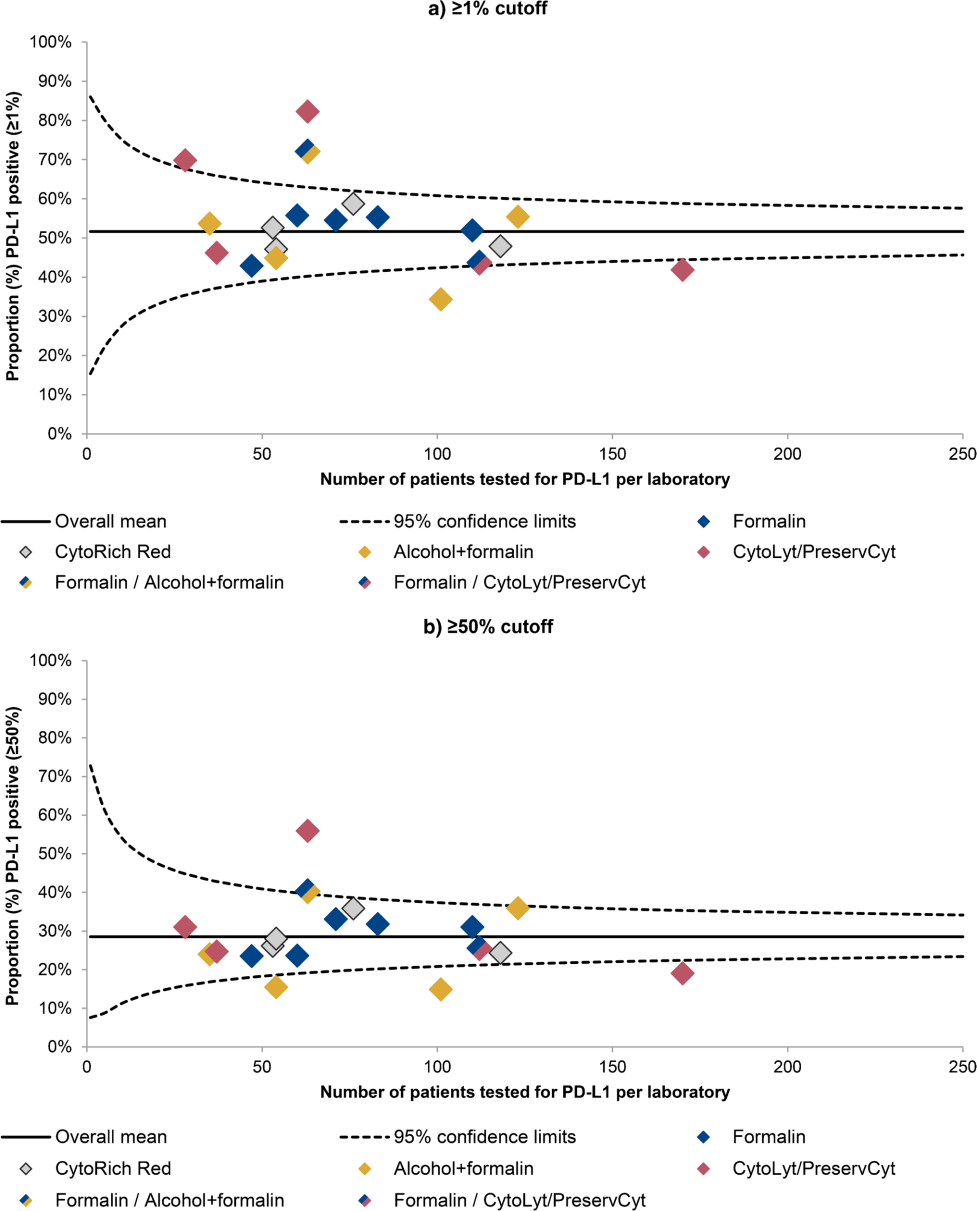
Funnel plots showing interlaboratory variation in programmed death ligand-1 (PD-L1) positivity, with PD-L1 positivity rates adjusted for case mix including the variables fixative and cell block method. PD-L1 positivity was determined using either a 1% cutoff (**a**) or a 50% cutoff (**b**). For each laboratory, case mix-adjusted positivity rates are displayed against the total number of patients tested for PD-L1 (diamonds). The color of the diamonds indicates the fixative that was used the most in each laboratory (see legend). The black line shows the overall mean proportion of PD-L1 positive patients, surrounded by its 95% confidence limits (dotted lines)

When the variables fixative and CB method were included in the multivariable logistic regression models for case mix correction, the number of laboratories falling outside the 95% confidence limits decreased from eight (42.1%) to five (26.3%) at the 1% cutoff (Fig. 4a). Adding the variables fixative and CB method to the case mix correction analysis resulted in a maximum variation between PD-L1 positivity rates of 47.9% (34.4–82.3%). This was a slightly wider range than the range of PD-L1 positivity rates adjusted for case mix without fixative and CB method, which was 46.4%. This seems mainly driven by a single laboratory that went from falling within the 95% confidence limits when adjusted for case mix without the variables fixative and CB method, to falling far outside the upper 95% confidence limit when adjusted for case mix including fixative and CB method.

At the 50% cutoff, adjusting PD-L1 positivity rates for case mix with the inclusion of the variables fixative and CB method again resulted in a decrease of the number of laboratories differing significantly from the mean from eight (42.1%) to five (26.3%) (Fig. 4b). The PD-L1 positivity rates of the individual laboratories ranged from 14.9 to 55.9%, resulting in a maximum variation of 41.0%. This maximum variation was wider than the maximum variation of PD-L1 positivity rates adjusted for the case mix without the fixative and CB method (41.0% vs. 31.0%). Again, this seems mainly driven by the same outlier laboratory as at the 1% cutoff.

At both cutoffs, adding the variables fixative and CB method to the multivariable logistic regression model resulted in a lower log-likelihood value in comparison to the model without the variables fixative and CB method, indicating a better fit to the data of the extended model. The difference in fit between the models was statistically significant based on the LRT (*p*-value < 0.01).

## Discussion

In this cohort study based on real-world data, the variation in fixation and CB processing of cytology samples by pathology laboratories in the Netherlands was assessed. We revealed that many differences exist in both the use of fixatives and of CB methods, sometimes including multiple methods within one laboratory. Correcting PD-L1 positivity rates of individual laboratories for differences in the use of fixative and CB method resulted in a reduction of the number of laboratories that differed significantly from the mean PD-L1 positivity. Moreover, the observed decrease in interlaboratory variation was considerably greater than the decrease that was seen when PD-L1 positivity rates were corrected for differences in patient and sample characteristics without the variables fixative and CB method.

First of all, the amount of different fixation and processing methods and reported combinations among the laboratories that responded to our survey is enormous: Within a total of 28 laboratories, 19 different combinations of fixation and processing cytological material into a CB could be discerned. These results are comparable to those of other studies that used surveys to assess interlaboratory variation in both fixation and CB methods, which also showed large amounts of variation in cytology processing methods between laboratories [8, 22–24].

Both methanol-based and ethanol-based fixatives have a potentially deleterious effect on PD-L1 immunostaining performed on CBs [15–17], with a risk of false-negative PD-L1 immunostaining results. Indeed, correcting for differences in cytology fixation and CB processing methods between laboratories resulted in a reduction in the number of laboratories differing significantly from the mean in PD-L1 positivity. Formalin post-fixation may reverse the negative effects of alcohol fixation to some degree [15], with some studies showing good concordance in PD-L1 positivity between histology and cytological specimens from the same tumor fixed in an alcohol-based fixative followed by formalin fixation [25–27]. It is unclear, however, what the maximum duration of alcohol fixation is after which formalin post-fixation is still effective, and what the most optimal formalin post-fixation time would be. CytoRich Red, containing both alcohols and formaldehyde, did not seem to have a negative effect on PD-L1 immunostaining in various studies [15, 26, 28]. Likewise, univariable logistic regression analyses in our study did not show a statistically significant difference in the odds of finding PD-L1 positivity between CytoRich Red fixation and formalin fixation, while the odds of scoring PD-L1 as positive were significantly lower in samples fixed in CytoLyt/PreservCyt without formalin post-fixation or in alcohol with formalin post-fixation compared with samples fixed in formalin.

It is possible that differences in the CB method also influence interlaboratory variation in PD-L1 positivity, regardless of the fixation method. Yet, very few studies have been published that assessed the influence of the CB method on immunostaining independently of the fixation method. In a study by Lloyd et al. [17], cytology samples processed into CBs with the Cellient-automated CB system showed optimal PD-L1 staining results compared with CB preparation according to the plasma-thromboplastin method. However, the authors advise against the use of CytoLyt as a collection medium due to the poor performance of PD-L1 immunostaining in samples collected in CytoLyt. Remarkably, CytoLyt is the collection medium of choice recommended for use with the Cellient system by the manufacturer. In our study, nearly 75% of the Cellient processed samples were fixed in CytoLyt/PreservCyt, and the remainder were fixed in an alcohol-based fixative with formalin post-fixation. None of the Cellient processed samples were fixed in formalin only. All in all, based on the available literature, it is very likely that the influence of differences in cytology processing methods on interlaboratory variation in PD-L1 positivity can be attributed mostly to differences in fixation methods.

We have reported previously that a large degree of variation in PD-L1 positivity between laboratories is problematic. Indeed, this could result in patients receiving different PD-L1 test results depending on the pathology laboratory where their material is tested [18]. Thus, efforts should be taken to keep interlaboratory variation in PD-L1 positivity to a minimum. Based on the current study, an important step to take would be to create more uniformity between laboratories in the way that cytology samples are fixed and processed, using a method that does not negatively influence immunostaining results. This desire for uniformity has been expressed by others [8, 29], too, and could prove beneficial not only to results from PD-L1 immunostaining but also to results from other immunohistochemical assays that show adverse effects of alcohol fixation, such as for progesterone receptor [30] and MIB1 [31]. External quality assessment (EQA) schemes specifically designed to assess immunocytochemistry could perhaps aid in uncovering possible technical issues and in promoting standardization [32]. Future studies should investigate which method is the preferred (combination of) cytology processing method(s) for PD-L1 testing.

Potentially, rigorous validation and optimization of immunostaining protocols that are used on cytology samples but have originally been validated on FFPE tissue samples could aid in diminishing variation as well. Unfortunately, it has been shown that validation and optimization of immunostaining protocols for cytology samples are not common practice [23, 24], even though organizations such as the College of American Pathologists (CAP) recommend that a sufficient number of cases should be tested to ensure that immunohistochemical assays achieve similar results when performed on cytological material compared to histological material [33]. No advice is given, however, on the criteria and number of specimens needed for validation, and it is stated that “separate validation of all markers on all potential cytologic specimens is generally not feasible” [33]. The type of material that should be used for validation may often not be clear either, or it may be difficult to collect enough material, especially when dealing with small cytology samples. On top of that, some laboratories receive cytological specimens from external laboratories for immunocytochemical testing, which may have been fixed and processed in a variety of ways. Moreover, with PD-L1 immunostaining, a decrease in staining intensity could result in false-negative staining results, but it is hard to determine what level of decrease in staining can still be accepted and what level would actually cause problems in clinical practice. All these factors may complicate the proper validation and optimization of PD-L1 immunohistochemical stains that are used on cytological specimens. On top of that, laboratories may use commercial assays for PD-L1 immunostaining, which use standardized protocols developed by the manufacturer that cannot simply be adjusted.

After correction for the case mix including variation in the fixative and CB method, the amount of interlaboratory variation in PD-L1 positivity was still substantial at both cutoffs. Compared to histology, tissue architecture is disrupted in cytology samples, which can complicate the recognition of tumor cells. Also, it may be a lot harder to distinguish tumor cells from inflammatory cells, especially macrophages, which may lie adjacent to or intermixed with isolated tumor cells [34, 35]. The level of experience and training of the pathologists scoring PD-L1 immunostaining on cytology in a routine clinical pathology setting may not be the same in all laboratories, all the more so because scoring of PD-L1 on cytology requires adequate training in both cytopathology and PD-L1 scoring. Structural differences between laboratories could arise, for instance, when inflammatory cells are often mistaken for tumor cells. Moreover, small tissue samples can lead to an underestimation of PD-L1 expression [36], which probably also applies to cytology samples. In fact, it has been shown that PD-L1 staining results of CBs and resection specimens are more concordant when a greater number of tumor cells were present in the CB [37]. Perhaps laboratories that structurally receive cytological samples that contain more tumor cells, for instance, because multiple passes or bigger needles are used to collect material, have higher PD-L1 positivity rates based on cytology than other laboratories. Our study, however, does not provide the data to properly investigate this hypothesis.

Of note, in our study, an association was found between sex and PD-L1 expression, with PD-L1 positivity being more likely in samples from women than from men (Table 1). While similar results have been shown by others [38, 39], various other studies did not find any association between PD-L1 expression and sex [40–45] or found that PD-L1 was more likely to be positive in men than in women [46–48]. These studies, however, primarily used FFPE material, mostly from surgical resections or biopsies. Our study only included cytological samples, many of which were not fixed in formalin or embedded in paraffin, which might explain the differences in results. Similarly, while some studies did not find a statistically significant association between PD-L1 expression and sampling site [49, 50], comparable to our results (Table 1), others showed that pleural and nodal metastases were more likely to express PD-L1 than primary tumors [42]. Again, the difference in results could potentially be explained by the latter study using FFPE material which largely came from biopsies or surgical resections, while our study only used cytological material fixed and processed in a variety of ways. Moreover, the differences in proportions between PD-L1 positivity and negativity of the various characteristics in Table 1 were tested through univariate analysis, which does not account for potential confounding factors. Also, since we used a large cohort in our study, small and maybe even clinically insignificant differences might be statistically significant, whereas they might not have been in studies with smaller sample sizes. These factors should be taken into account when interpreting these results.

This study has some limitations. Most importantly, even though a considerable amount of laboratories responded to our survey, we did not receive answers from all laboratories. This resulted in the exclusion of 516 patients from the PALGA data set, for whom the fixative and CB method were unknown. Given the current variation in the fixation and CB methods, it is to be expected that the overall number of methods used would only be larger, potentially resulting in a larger baseline variation among laboratories to start with. This should be considered when interpreting the analyses of the influence of variation in fixation and CB processing on interlaboratory variation in PD-L1 positivity. Second, the respondents reported varying mean fixation times. Vigliar et al. [51] showed that formalin fixation time influences PD-L1 immunostaining results on CBs. This could be the case with other fixatives, too. Unfortunately, we did not know the fixation times for individual samples in the PALGA data set and, thus, could not incorporate information on fixation time in our analyses. Third, due to a large amount of variation in cytology processing methods, especially in fixation methods, it was quite difficult to divide these various methods into larger categories. In fact, if numbers had allowed to include all methods as they were, a better correction could have been performed. However, we do feel that the distribution that we used is compatible with the currently available literature. Finally, since our study is based on real-world pathology data, we were dependent on the way that pathologists report their findings. For instance, while it would have been interesting to include an analysis of TPS on a continuous scale, the fact that various laboratories only reported TPS in categories did not allow us to do so. Also, some potentially relevant information, such as information on previous treatment, is not regularly part of pathology reports, meaning that we could not correct for potential differences between laboratories within these areas. Regarding treatment status, however, in many patients, PD-L1 testing was performed on the initial diagnostic material, either at the time of diagnosis or at a later time. We also excluded patients with more than one primary lung tumor, to avoid including data from PD-L1 tests that might have been influenced by previous treatment. We therefore expect the number of patients in which PD-L1 testing was performed solely on material collected after administration of chemotherapy to be too small to influence our results in a significant way.

To conclude, this study shows that a lot of variation exists between laboratories in the methods used for fixation and CB processing of cytological samples. We have demonstrated that these differences influence interlaboratory variation in PD-L1 positivity in NSCLC patients, with a decrease in the amount of variation when PD-L1 positivity rates are corrected for differences in fixation and CB methods. A high degree of variation in PD-L1 positivity between laboratories is problematic, because this will almost inevitably lead to patients receiving different courses of treatment depending on the laboratory where their cytological material is stained and scored for PD-L1. These results warrant the need for more research to determine the best methods of fixation and CB processing of cytology samples on which PD-L1 immunostaining is to be performed, and for harmonization of these methods between laboratories.

## Supplementary Information

Below is the link to the electronic supplementary material.

Supplementary file1 (PDF 247 KB)

## Data Availability

The data that support the findings of this study are available from PALGA, but restrictions apply to the availability of these data, which were used under license for the current study, and so are not publicly available. Data are however available from the authors upon reasonable request and with permission of PALGA.
