# Supplementary material for: Nationwide differences in cytology fixation and processing methods and their impact on interlaboratory variation in PD-L1 positivity
Source: Virchows Arch. 2022 Nov 12;482(4):707–20. doi: 10.1007/s00428-022-03446-w (PMC10067664; doi:10.1007/s00428-022-03446-w)
Supplement: Supplementary file 1 — Supplementary file1 (PDF 247 KB) [file 428_2022_3446_MOESM1_ESM.pdf]

SUPPLEMENTARY DATA

Virchows Archiv

Nationwide differences in cytology fixation and processing methods and their impact on interlaboratory variation in PD-L1 positivity

B.M. Koomen, M. de Boer, C. van Dooijeweert, A.S.R. van Lindert, I.A.G. Deckers, Q.J.M. Voorham, S.M. Willems

Corresponding author: Bregje M. Koomen (b.m.koomen@umcutrecht.nl), department of Pathology, University Medical Center Utrecht, Utrecht University, Heidelberglaan 100, 3584 CX, Utrecht, the Netherlands.

Supplementary information 1

Questionnaire concerning fixation and cell block processing methods of cytological material used for PD-L1 immunostaining in NSCLC patients

1. Is PD-L1 immunostaining for NSCLC patients performed in your own laboratory or in an external laboratory?
   - o Own laboratory
   - o External laboratory
   - o Other, namely: …

2. How many ways of processing cytology samples before performance of PD-L1 immunostaining are used within your laboratory? (e.g. different methods for pleural effusions and fine needle aspirations (FNA))
   - o 1 way
   - o 2 ways
   - o 3 ways
   - o Other, namely: …

In case of >1 method, respondents were asked to answer question 3-8 for each method separately.

3. For which type of material are you answering the following questions? (e.g. pleural effusions, FNA, bronchial lavage)
…

4. What medium is the cytological material collected in? (please be as specific as you can)
…

5. Which fixative is used? (please be as specific as you can)
…

6. What is the estimated fixation time?
   Minimum: …
   Maximum: …
   Mean: …

7. Which intermediate steps are used, if any? (e.g. post-fixation, rinse step, etcetera)
…

8. Which method is used to create a cell block? (please be as specific as you can)
…

Supplementary fig. 1

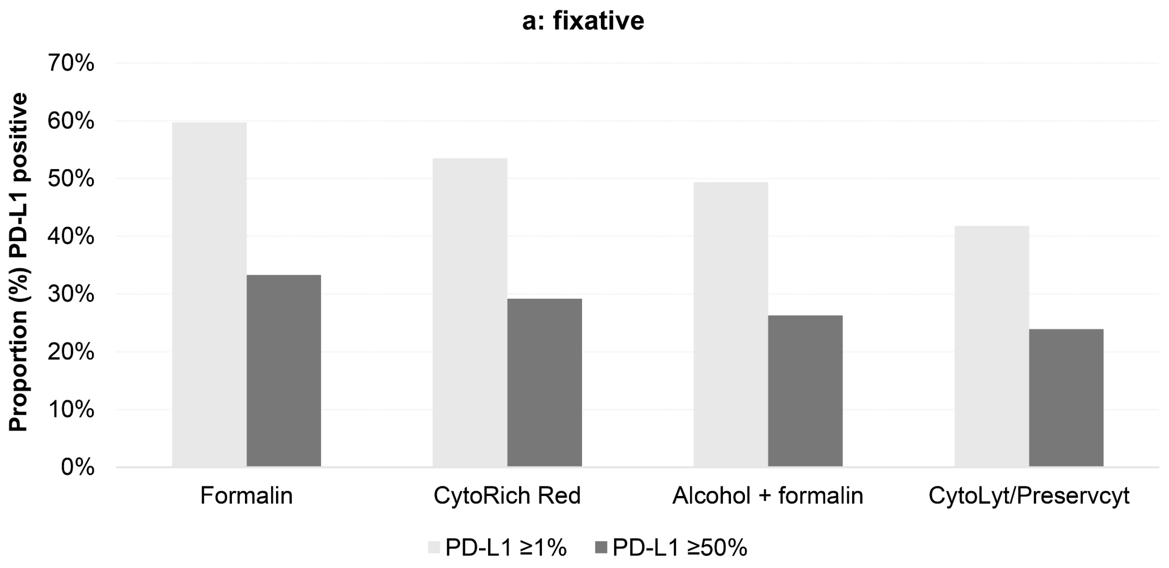

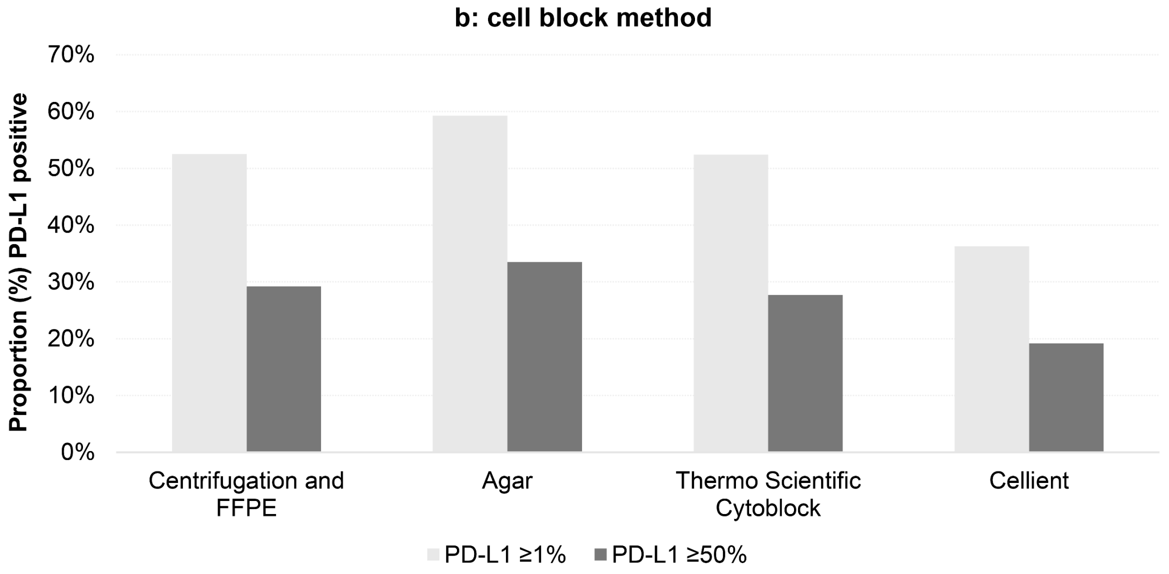

Mean PD-L1 positivity rates per fixative (**a**) and per cell block method (**b**). PD-L1 positivity is determined using either a ≥1% cutoff or a ≥50% cutoff. Abbreviations: FFPE = formalin-fixed paraffin-embedded; PD-L1 = programmed death ligand-1.
